# Molecular screening of the *CYP4V2* gene in Bietti crystalline dystrophy that is associated with choroidal neovascularization

**Published:** 2011-07-20

**Authors:** Gandra Mamatha, Vetrivel Umashankar, Nachiappan Kasinathan, Tandava Krishnan, Ravichandran Sathyabaarathi, Thirumalai Karthiyayini, John Amali, Chetan Rao, Jagadeesan Madhavan

**Affiliations:** 1 SN ONGC Department of Genetics and Molecular Biology, Vision Research Foundation, Sankara Nethralaya, Chennai, India; 2 Centre for Bioinformatics, Vision Research Foundation, Sankara Nethralaya, Chennai, India; 3 Shri Bhagawan Mahavir Vitreoretinal Services, Medical Research Foundation, Sankara Nethralaya, Chennai, India

## Abstract

**Purpose:**

Bietti crystalline dystrophy (BCD) is an autosomal recessive disease characterized by intraretinal deposits of multiple small crystals, with or without associated crystal deposits in the cornea. The disease is caused by mutation in the cytochrome p450, family 4, subfamily v, polypeptide 2 *(CYP4V2)* gene. Choroidal neovascularization (CNV) is a rare event in BCD. We report two cases of BCD associated with CNV. *CYP4V2* and exon 5 of tissue inhibitor of metalloproteinase 3 *(TIMP3)* were screened in both cases. A patient with BCD, but without CNV, was also screened to identify pathogenic variations.

**Methods:**

Three BCD families of Asian Indian origin were recruited after a comprehensive ophthalmic examination. Genomic DNA was isolated from blood leukocytes, and coding exons and flanking introns of *CYP4V2* and exon 5 of *TIMP3* were amplified via polymerase chain reaction (PCR) and were sequenced. Family segregation, control screening, and bioinformatics tools were used to assess the pathogenicity of the novel variations.

**Results:**

Of the three BCD patients, two had parafoveal CNV. The patient with BCD, but without CNV had novel single base-pair duplication (c.1062_1063dupA). This mutation results in a structurally defective and unstable protein with impaired protein function. Four novel benign variations (three in exons and one in an intron) were observed in the cohort. Screening of exon 5 of *TIMP3* did not reveal any variation in these families.

**Conclusions:**

A novel mutation was found in a patient with BCD but without CNV, while patients with BCD and CNV did not show any pathogenic variation. The modifier role of *TIMP3* in the pathogenesis of CNV in BCD was partly ruled out, as no variation was observed in exon 5 of the gene. A larger BCD cohort with CNV needs to be studied and screened to understand the genetics of CNV in BCD.

## Introduction

Bietti crystalline dystrophy (BCD) is characterized by multiple small crystalline intra-retinal deposits throughout the posterior pole of the retina, with or without perilimbal subepithelial crystal deposits in the cornea. This autosomal recessive inherited disease progresses with retinal pigmental epithelium (RPE) and choroidal atrophy. Most patients develop nyctalopia and paracentral scotoma, generally by the third decade of life. Central vision is affected during the late stages of BCD, but sudden central visual loss is atypical. Even though the disease has been reported among Caucasian populations, it is more commonly described among East Asians, with a genetic frequency of 0.005 [1].

Bietti crystalline dystrophy is caused by mutations in the cytochrome p450, family 4, subfamily v, polypeptide 2 *(CYP4V2)* gene, which belongs to the cytochrome P450 family of genes in chromosome 4q35 [2]. The disease is characterized by reduced conversion of fatty acid precursors into n-3 polyunsaturated fatty acid (n-3 PUFA). This is due to a deregulation in lipid metabolism through deficient lipid binding, elongation, or desaturation [3]. The characteristic crystals of BCD, which resemble cholesterol or cholesterol esters when observed in the retina, can also be found in the cornea, conjunctiva, fibroblasts, and in circulating lymphocytes. Defective lipid metabolism ultimately leads to retinal degeneration and choroidal sclerosis. Histopathologically, the manifestations of BCD include extensive evidence of advanced chorioretinal atrophy characterized by the generalized loss of and sclerosis of the choriocapillaris, with crystals in the choroidal fibroblasts [4].

Choroidal neovascularization (CNV) is usually associated with defects of the retinal pigment epithelium (RPE) and Bruch’s membrane; these are also commonly seen in age-related macular degeneration, which is a disease of complex etiology. CNV is unusual in hereditary retinal degeneration with the exception of a few conditions, such as Sorsby fundus dystrophy, which has a higher incidence of CNV than other hereditary retinal disorders due to defects in of tissue inhibitor of metalloproteinase 3 *(TIMP3;* an inhibitor of matrix metalloproteinases [MMPs] that regulates extracellular matrix formation) [5]. The TIMP3 protein is secreted by RPE and is a component of Bruch’s membrane.

There are three documented case reports of BCD associated with CNV [6-8]. Genotype characterization was not performed to identify the genetic variations in these case reports. In the present study, we report on two patients with BCD and subfoveal choroidal neovascularization with *CYP4V2* screening results. A separate patient with BCD but without CNV was screened, leading to identification of a novel pathogenic variation. Furthermore, we screened for exon 5 of *TIMP3* to understand its role as a modifier that could induce CNV in BCD.

## Methods

### Patients

Three patients with a history of BCD and of Asian Indian origin were recruited from the medical research foundation of Sankara Nethralaya, a not-for-profit charitable eye hospital in Chennai, India. The patients were evaluated using a Snellen chart, slit-lamp biomicroscopy, and indirect ophthalmoscopy. Subsequent to the initial evaluation, further investigations were performed with techniques including Humphrey automated perimetry, electroretinography (ERG), fundus fluorescein angiography (FFA), and optical coherence tomography (OCT). Diagnosis of BCD was based on the presentation of primary features including glittering crystalline deposits in the posterior pole of the retina (associated with degeneration of RPE) and perilimbal subepithelial crystal deposits in the cornea. The study was conducted in accordance with the tenets of the Declaration of Helsinki.

### *CYP4V2* and *TIMP3* mutation analysis

Genomic DNA was isolated from blood leukocytes using a QIAamp DNA blood maxi kit columns (Genetix, Chennai, India). Coding exons and flanking intronic regions of *CYP4V2* (GenBank NM_207352.3) and exon 5 of *TIMP3* (GenBank NM_000362.4) were amplified via PCR and sequenced (ABI 3100 Avant, Applied Biosystems, Lab India, Chennai) at the Vision Research Foundation, Sankara Nethralaya. Any changes observed were confirmed to be bidirectional. If blood samples of the affected individual’s family members were available, segregation of the variation identified in the affected individual was assessed in the family. Novel mutations were screened for using sequencing with a control panel of 138 chromosomes. All primers and conditions are listed in Table 1.

**Table 1:** Coding exons and flanking intronic regions of *CYP4V2* and exon 5 of *TIMP3* primers and conditions.

**Exon number**	**Primer sequence (5′-3′)**	**Annealing temperature (°C)**
Exon 1	F: CAACCTCGCAGCACCCTCAGAA	62
	R: ACTTTGGGATGGGGCACTAGCAGT	
Exon 2	F: ACCTGGCTTCCTCTAACAGTAACA	60
	R: TTTTTGTGCTGAAATGGCTGAA	
Exon 3	F: AGATTCGCCTCCTCCCACCTCAC	67
	R: ACCTGGACTCTTGGCCTCTTGACG	
Exon 4	F: TGCCAAAAGCATTTGAGAACCTGT	63
	R: CGCGCTGAAGAGCCCGTCAC	
Exon 5	FPAGGAAGAACAGGAACAGGGAGTAG	63
	R: CAACGCAGAAATTGTTAGCAATAA	
Exon 6	F: GCTTCATGGGATGCGTAATAGC	60
	R: GAAATGAACGGTGGGGATGGT	
Exon 7	F: CCTATGTTGTCGAAATGTTGAAAT	52*
	R: TCTGAAGAAGTTGAGCTGGTACTT	
Exon 8	F: TTGCAGTCACAGTGCAGTCATCA	67
	R: CCAGCATCCGGCCTAGTACAGTC	
Exon 9&10	F: ATGCCATGCCTTGATCCACCTGT	63
	R: TGGGCAATGTCACATCACATCTCA	
Exon 11	F: CTCTTCATCTTTAACAGGTGTTCC	65
	R: CAAAACTCAAAACTTTTTCTTTGT	

Abbreviations: F represents forward primer, R represents reverse primer, asterisk represents touchdown- 58 °C to 52 °C (15 cycles); 52 °C (20 cycles).

### Analysis of *CYP4V2* variations by structural bioinformatics; molecular modeling

The sequence information of human P450 4V2 was retrieved from the Swiss-Prot database (GenPept Q6ZWL3). The three-dimensional (3D) structure for human CYP4V2 is yet to be elucidated, hence the structure was modeled using mammalian CYP2C5 (PDB ID: 1DT6) and bacterial CYPBM3 (PDB ID: 2HPD) as templates and using Modeler 9v7 (University of California, San Francisco, laboratory of Andrej Sali, University of California, San Francisco, CA), a molecular modeling suite [9]. Since heme is a cofactor of CYP4V2, the heme atom was positioned in the model using an align-ligand module with heme-bound CYPBM3 as a template. A stringent molecular modeling procedure was followed for the structure generation of the missense mutation Q259K (MT1) and the frame-shift mutation p. Val354Serfs2X (MT2) with wild type CYP4V2 (WT) as a template. Furthermore, the predicted structures for the WT and mutants MT1 and MT2 were refined using the GROMACS 3.3.2 software package (Uppsala University, Sweden) [10] with an extended version of the GROMOS97 force field. The generated models were also optimized by energy minimization for 500 steps of steepest descent in the presence of solvent to fix the steric clashes between atoms. The optimized structures were further validated for their stereochemical properties using Procheck (Laboratory of Dr. Roman A. Laskowski, European Bioinformatics Institute, Hinxton, UK) [11] by measuring the Phi-Psi angles using a Ramachandran plot. The quality of the predicted structures was validated based on LG-score and MaxSub values scored by a ProQ server (Stockholm Bioinformatics Center, Stockholm, Sweden), a tool for protein structure quality prediction [12].

### Mutational analysis based on sequence and structural features

At the protein sequence level, based on the multiple sequence alignment with the close homologs of CYP4V2, the significance of the mutated residues was identified using ConSurf server (Laboratory of Dr. Nir Ben-Tal, Department of Biochemistry and Molecular Biology, Tel Aviv University, Tel Aviv, Israel) [13] with the default values, except for the multiple sequence alignment using CLUSTALW (Laboratory of Dr. David G. Dineen, Complex and Adaptive Systems Laboratory [CASL], University College Dublin, Belfield, Ireland) [14]. Blosum70 (Blocks Substitution Matrix 70; Laboratory of Dr. Steven Henikoff, Howard Hughes Medical Institute, Fred Hutchinson Cancer Research Center, Seattle, WA) [15] was used to assess the impact of the substitutive mutations studied. Since the rate of folding plays a crucial role in protein function, and is governed by the physicochemical properties conferred by the composition of the amino acids, the folding rate of WT, MT1, and MT2 were predicted using the FOLD-RATE server (Laboratory of Dr. M. Michael Gromiha, Computational Biology Research Center [CBRC], National Institute of Advanced Industrial Science and Technology, Tokyo, Japan) [16]. Moreover, the variation in the protein stability due to atomic contact energy was also predicted for the generated structures using RankViaContact (Laboratory of Dr. Mauno Vihinen, University of Tampere, Finland) [17]. STRIDE (Structural Identification, Laboratory of Dr. Dmitrij Frishman, Department of Genome Oriented Bioinformatics, Technical University of Munich, Germany) [18] was used to predict the secondary structural elements formed by each of the residues for the structures generated, and thereby determined the conformational variation acquired due to the mutations. As the electrostatic potential of proteins plays an important role in molecular interactions, it was calculated using online APBS server (Laboratory of Dr. Nathan A. Baker, Departments of Chemistry and Biochemistry, Washington University, Seattle, WA) [19] by solving the Poisson-Boltzmann equation and the variations were visualized using Jmol (Laboratory of Dr. Robert M. Hanso, Department of Chemistry, St Olaf College, Northfield, MN). The phenotypic variation and the functional effects of the mutation were also analyzed using scale-invariant feature transform (SIFT) and polymorphism phenotyping (PolyPhen).

## Results

### Clinical features

**Figure 1:**
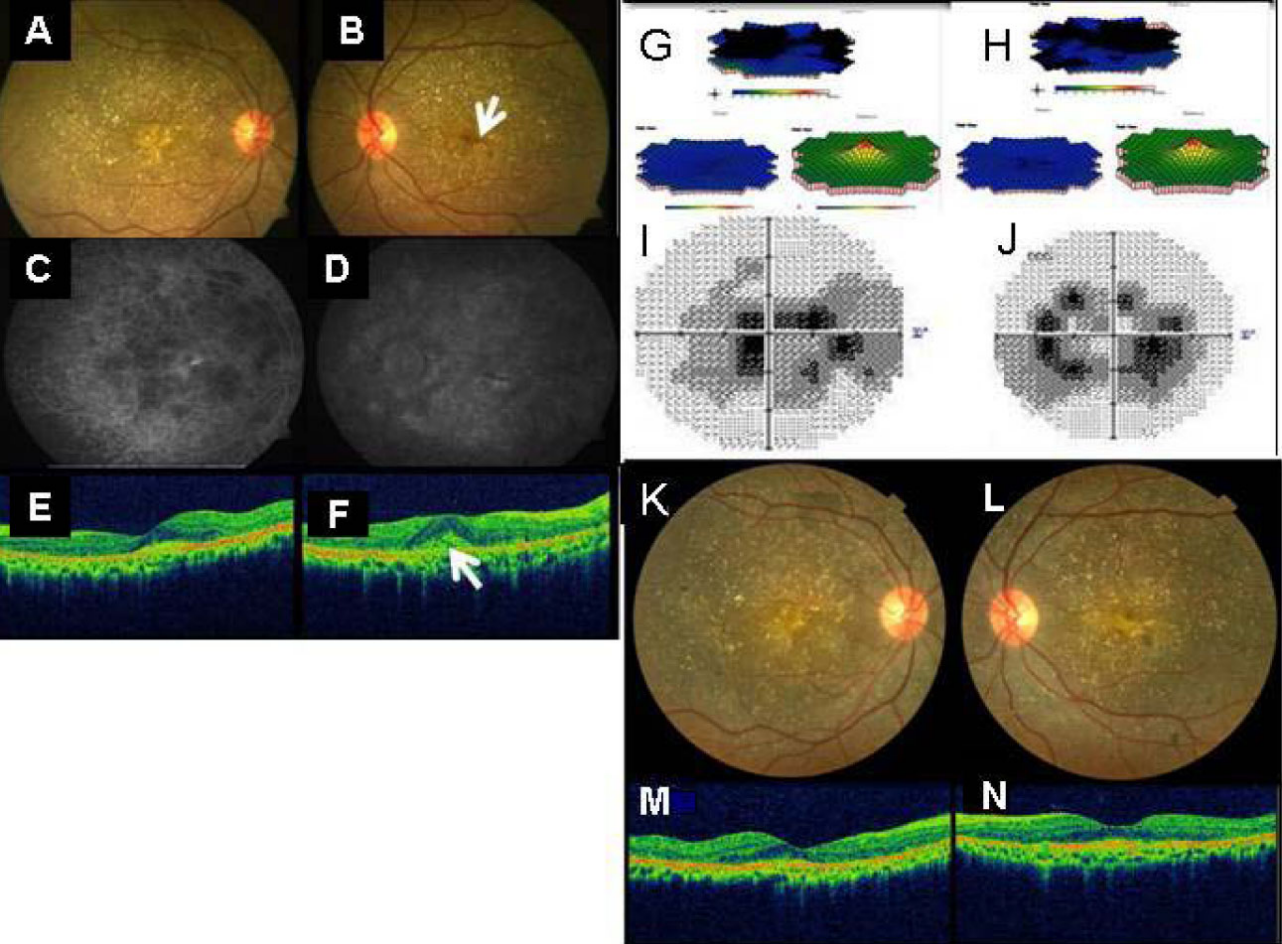
Clinical and investigation pictures of patient 1. **A**: Color fundus photograph of right eye showing scarred choroidal neovascularization (CNV). **B**: Pretreatment color fundus photograph of the left eye showing retinal crystals with active CNV (arrow) and sub retinal hemorrhage. **C**: Fundus fluorescein angiography (FFA) of right eye shows staining. **D**: FFA of the left eye shows areas of window defects corresponding to areas of RPE and choriocapillaris atrophy with blocked fluorescence along the areas of crystalline deposits and subretinal hemorrhage. Hyperfluorescence spot (leaked fluorescein) is seen on right eye subfoveal region, suggestive of active CNV. **E**: SD-OCT of right eye showed foveal contour with scarred subfoveal CNV. **F**: SD-OCT of left eye showed loss of foveal contour with increased retinal thickness with an active sub foveal CNV (arrow). **G**, **H**: Multifocal ERG revealed grossly reduced central and paracentral ring responses with reduced peripheral ring response in both eyes. **I**, **J**: Visual field defects were also noted on Humphrey visual field perimetry. **K**, **M**: Color fundus photo and OCT of right eye at last follow-up. **L**: Post Ranibizumab treated color fundus photograph of left eye showed scarred CNV as confirmed by OCT (**N**) at last follow-up.

Two patients in our cohort presented with BCD and CNV (Table 2). The first patient, a 31-year-old male, stated that he had experienced a decrease in central vision in both eyes during the past year, with associated difficulty in night vision. There was no history of similar eye problems in the family. Upon examination, he had a best-corrected Snellen visual acuity of 6/9 in both eyes. The anterior segment examination showed paralimbal crystals in both eyes. Fundus examination revealed diffuse RPE alterations, with the numerous intraretinal crystalline deposits in both eyes suggestive of BCD. A scarred subfoveal CNV was noted in the right eye (Figure 1A). Subretinal hemorrhage with active CNV was noted in the subfoveal region of the left eye (Figure 1B). Spectral domain optical-adherence tomography (SD OCT; Cirrus, Carl Zeiss Meditec, Inc., Dublin, CA) revealed the presence of high-reflective intraretinal deposits in both eyes; the left eye had subfoveal CNV with increased retinal thickness and moderately reflective echoes suggestive of hemorrhage (Figure 1E,F). Full-field ERG showed waveforms of normal amplitude and implicit time for both photopic and scotopic responses, while multifocal ERG revealed a reduced central, paracentral, and peripheral ring response for both amplitude and implicit time in both eyes that was different than expected (based on normal reference points) given the individual’s age (Figure 1G,H). Fundus fluorescein angiography of right eye and left eye shows areas of window defects corresponding to areas of RPE and choriocapillaris atrophy with blocked fluorescence along the areas of crystalline deposits and subretinal fluid in the left eye. Right eye also showed staining suggestive of scarred CNV and leakage in the left eye suggestive of active CNV (Figure 1C,D). The patient’s serum triglyceride level was found to be 171 mg/dl (normal range, 60 to 165 mg/dl). The nature of BCD with CNV was explained to the patient and he was offered a treatment option of anti-vascular endothelial growth factor (anti-VEGF) injection after its roles and limitations were explained. The patient consented to this treatment and was administered three injections of 0.05 mg ranibizumab (Lucentis^®^; Novartis Pharma AG, Basel, Switzerland; Genentech Inc., South San Francisco, CA), intravitreally at monthly intervals. At the last follow up, six months after the final injection, the patient maintained the same visual acuity. Fundus examination showed scarring from CNV in both eyes (Figure 1K,L) that was confirmed on OCT (Figure 1M,N).

**Table 2:** Clinical Presentation of Bietti crystalline dystrophy (BCD) patients.

**Case**	**Age/** **gender**	**Visual acuity logmar**		**Visual field**		**CCD**	**RCD**	**ERG**	
		**OD**	**OS**	**Central**	**Periphery**			**fF**	**mF**
1	31Y/M	0.2	0.2	Defective	Decreased	+	+*	Normal	Reduced in central ring
	32Y	0.2	0.2	Defective	Decreased	+	+	ND	ND
2	26Y/M	0.9	0.1	Defective	Decreased	NP	+	Normal	Reduced in central ring
	27Y	1	0.5	Defective	Decreased	NP	+*	ND	ND
3	21Y/F	0.2	0.2	Defective	Decreased	+	+	Reduced	Reduced in central ring

Abbreviations: CCD represents corneal crystalline deposits, RCD represents retinal crystalline deposits, ND represents not documented, NP represents not present, fF represents full field, mF represents multifocal, * CNV represents choroidal neavascularization noted.

The second patient was a 26-year-old male with diminished vision, of six months duration, in both eyes. His best-corrected visual acuity was 6/48 and 6/7.5 in his right and left eyes, respectively. The rest of the findings in this patient were similar to those in the first patient, except that the patient did not have crystal deposits in the cornea and had no sign of CNV. At the time of the patient’s one-year follow up visit, his visual acuity had significantly dropped to 6/60 in the right eye and 6/18 in the left eye. OCT revealed foveal scarring in the right eye with RPE pigmentation. The left eye revealed scarring of the choroidal neovascular membrane with a slight increase in retinal thickness.

In addition to these two patients, we included a third patient in the study, a female with BCD, but not CNV. The patient had diminished vision at night of 5 years duration. Her sister had the same problem in her medical history, but was not examined. The best-corrected visual acuity was 6/9 in both eyes. There were typical crystalline deposits in the posterior pole of the retina and in the supranasal portion of the cornea. Full-field ERG waveforms revealed a reduction in both amplitude and in implicit time for both photopic and scotopic responses in comparison to normal reference points for the woman’s age.

### *CYP4V2* and *TIMP3* analysis

Screening for *CYP4V2* in the patient with BCD without CNV revealed a novel mutation, a single base-pair duplication c.1062_1063dupA. None of the controls screened showed this variation. The variation resulted in the change of a non-polar hydrophobic amino acid valine to a neutral hydrophilic serine. For this base-pair duplication, two additional amino acids with different charges are replaced before undergoing truncation to produce an aberrant peptide (negatively charged hydrophilic aspartic acid is replaced by neutral hydrophilic glycine, and positively charged hydrophilic histidine is replaced by neutral hydrophilic serine). Genotyping *CYP4V2* in both patients with CNV identified three novel benign variations in the coding region without any functional significance. One of the novel benign variations c.775C>A resulted in the replacement of aminoacid glutamine with lysine. This variation was found in a homozygous or heterozygous state in 65% of the controls screened. No causative pathogenic variations were observed in *CYP4V2* in these two patients (Table 3). Screening exon 5 of *TIMP3* did not show a variation for any of the three patients.

**Table 3:** Mutation profile of *CYP4V2* in Bietti crystalline dystrophy (BCD) patient cohort.

**Gene**	**Cases**	**Location**	Nucleotide variation*	**Protein effect**	**Predicted pathogenic**
*CYP4V2*	1	Exon 4	c.453 A>C*	p.T151T (ht)	No
	1 & 2	Exon 6	c.775 C>A*	p.Q259K	No
	1 & 2	Exon 7	c.810 T>G	p.A270A	No
	1	Exon 7	c.987 G>A*	p.E329E	No
	3	Exon 8	c.1062_1063dupA*	p.Val354Serfs2X	Yes
	1, 2, & 3	Intron 9	IVS9–24 A>G*	Nil	No

* Novel variation; ht represents heterozygous.

### Bioinformatics prediction

**Figure 2:**
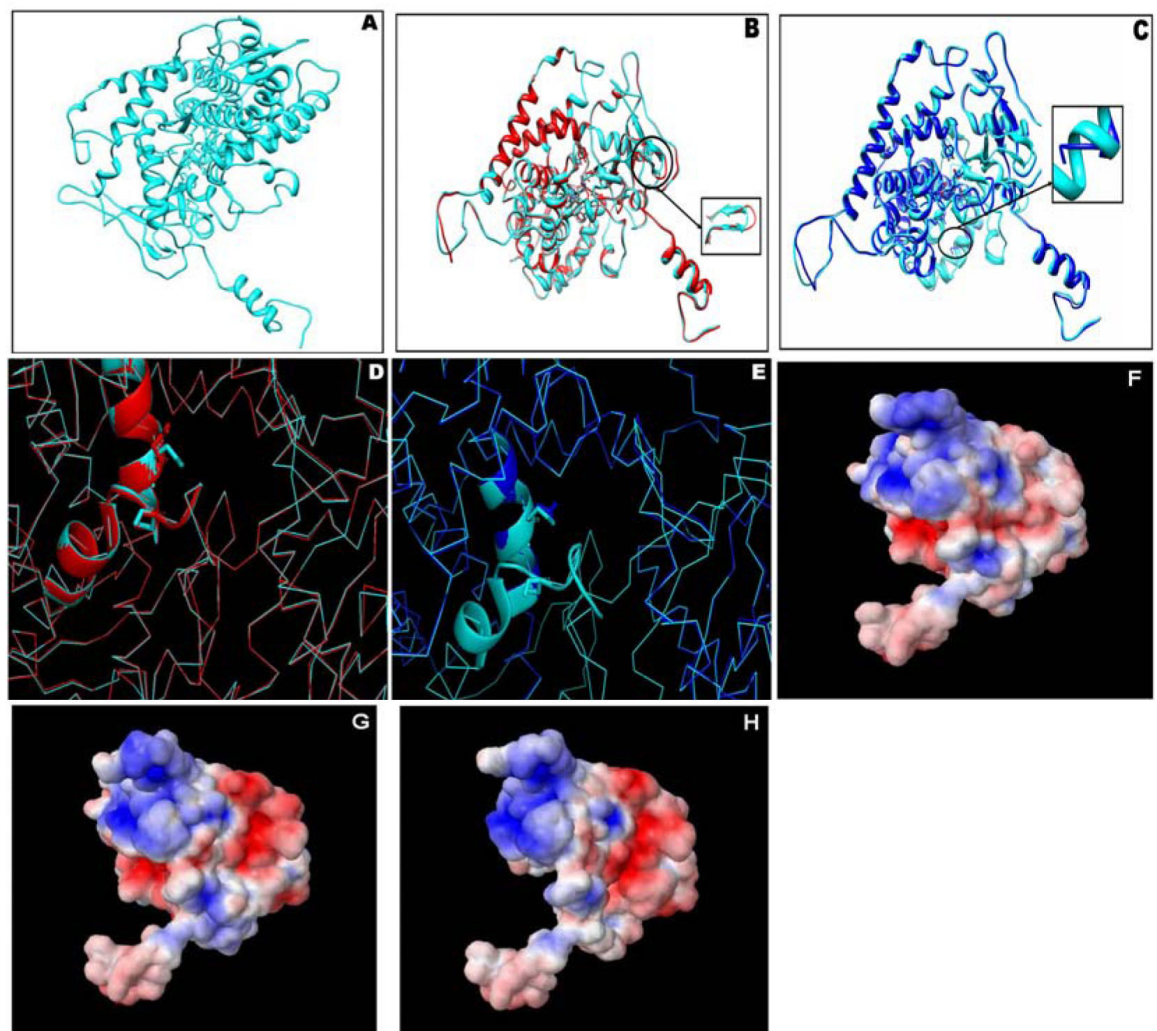
A structural analysis of CYP4V2 WT, MT1, and MT2. **A**: Homology model of WT- CYP4V2. **B**: Backbone superimposition of WT (cyan) with MT1 (Red), the altered secondary structures are shown in zoom view. **C**: Backbone superimposition of WT (cyan) with MT2 (Blue), the truncated region of MT2 in comparison with WT is shown in zoom view. **D**: Superimposed view of heme binding residues in WT (E329, C467) compared with MT1 (E329, C467). **E**: Superimposed view of heme binding residues in WT (E329, C467) compared with MT2 (E329). **F**, **G**, **H**: Electrostatic potential difference (scale: −2 to 2) in WT, MT1, and MT2, respectively. The blue color denotes a positive charge and red denotes a negative charge (the intensity of the shades are directly proportional to the charge levels).

The templates chosen for modeling CYP2C5 and CYPBM3 (with heme as a cofactor) sequences showed an overall identity of 25% and 29%, respectively, with WT-CYP4V2. Both CYP2C5 and CYPBM3 had a 44% similarity with WT-CYP4V2 and were modeled using Modeller9v7 (Laboratory of Dr. Andrej Sali). The predicted structure of WT was used as a template for modeling the structures of MT1 and MT2. MT1 and MT2 shared a 99% and 97% sequence identity with the WT, respectively, and were further refined using similar optimization protocols to those implemented for WT. No residues were found in the disallowed region of the Ramachandran plot for the generated structures. This confirms the plausibility of the generated models WT (Figure 2A), MT1 (superimposed view of WT with MT1; Figure 2B), and MT2 (superimposed view of WT with MT2; Figure 2C) of CYP4V2. The subtle conformational variation with the conversion of a few secondary structural elements in MT1 could confer only mild variation in the heme binding regions (Figure 2D) and may not affect the function of the protein. The truncation of an axial ligand-binding residue, cys467 in MT2 (Figure 2E), affects protein function by altering substrate binding. The normalized conservation scores for the mutant position in MT1 and MT2 were found to be 4 and 5, respectively. The blosum70 score generated using a substitution matrix for MT1 variant was found to be +1. This emphasizes the conservative nature, whereas in the case of MT2, a negative blosum score of −2 was observed, and thus predicts a defective mutant protein.

The predicted folding rate for WT was found to be 2.43/s, while for MT1 and MT2 it was found to be 3.65/s and −3.24/s, respectively, demonstrating that the MT2 mutation results in an unstable protein. The atomic contact energy of MT2 mutation was found to be highest with −243.405 J/mol, whereas for the WT and MT1 mutations the atomic contact energies were −510.510 J/mol and −534.050 J/mol, respectively. At the structural level, the backbone superimposition of MT1 with WT shows subtle conformational variation with the change in secondary structure elements at regions 406 to 408 and 411 to 414 in MT1 (Figure 2B). WT with MT2 superimposition shows a loss, due to truncation, of extended secondary structural elements from amino acid position 357 (Figure 2C). A significant variation was observed in the electrostatic potential at the heme-binding region of MT2 (Figure 2H), which may modulate the molecular interactions with its binding partners. In the case of MT1, it was observed that the variation at 259 with K is predicted to be tolerated with a SIFT score of 1.00, and is also benign as per polymorphism phenotyping, with a difference in position-specific independent counts (PSIC) of 0.442. Conversely, the phenotypic variation using SIFT with MT2 was found to be intolerant, with a score of 0.00. Polymorphism phenotyping also predicted that MT2 mutation would lead to impairment of protein function, with a PSIC difference of 2.095.

## Discussion

CNV is a rare event in BCD; to date, only three cases have been reported in the literature [6-8]. CNV and maculopathy have been reported in some RPE dystrophies, including Best vitelliform macular dystrophy, Sorsby pseudo-inflammatory macular dystrophy, and in North Carolina macular dystrophy [5,20-22]. To understand secondary CNV in patients with BCD, we genotyped *CYP4V2*, the primary gene that causes BCD.

In a patient with BCD, but without CNV, novel single nucleotide duplication in exon 8 of *CYP4V2* that result in a defective amino acid sequence (Val354Serfs2X) was seen. Bioinformatics tools were used to understand the effect of this mutation in the protein. The severity of the mutation was compared with another variation that results in amino acid replacement (Q259K). The negative Blosum score for Val354Serfs2X variation suggests a non-conservative substitution, which would affect heme binding and substrate interactions. The folding rate and atomic contact energy suggest that Val354Serfs2X variation is unstable in comparison to WT, while Q259K variation was as stable as the wild type. As per Swiss-Prot annotation for CYP4V2 (Q6ZWL3) entry, Glu329, and Cys467 are the heme-interacting residues. The heme binding cavity of the WT was superimposed with the respective residues of mutants: Q259K and Val354Serfs2X, to infer the change in the binding mode of heme. Studies of the electrostatic potential of mutations in Q259K variation (Figure 2G) and Val354Serfs2X variation (Figure 2H) infer that Val354Serfs2X variation might be pathogenic, as it showed significant variation in charge distribution that affected its intermolecular interactions. This prediction, more any other, indicates the instability of the Val354Serfs2X variation complex in comparison with Q259K variation and WT.

We were not able to identify the disease that caused a variation in *CYP4V2* in the BCD patient without CNV. The probable reasons for the inability to identify a variation in *CYP4V2* are: (i) the variation might have occurred in the promoter region of *CYP4V2*; (ii) it could be a heterogeneous disease in which additional genes of lipid metabolism are involved; and (iii) the disease pattern we have seen in two patients with CNV in our cohort simulates a phenotype similar to BCD. Exon 5 of the *TIMP3* gene, which codes for the C-terminal domain of the matrix metalloproteinase inhibitor protein is implicated in CNV in patients with Sorsby macular dystrophy and in some patients with age related macular degeneration (AMD). All of the known nucleotide variations detected so far in *TIMP3* have been identified in exon 5. Screening of exon 5 of *TIMP3* to understand its role as a genetic modifier showed no sequence variation that can be associated with the occurrence of CNV in these patients. We hypothesize that the chronic irritation of Bruch’s membrane by the crystals may have resulted in CNV in these patients; the contributing gene for this is yet to be identified. To conclude, more BCD patients with CNV need to be screened to understand the genetics and pathogenesis behind CNV in BCD.

## References

[1] Hu DN (1987). Prevalence and mode of inheritance of major genetic eye diseases in China.. J Med Genet.

[2] Li A, Jiao X, Munier FL, Schorderet DF, Yao W, Iwata F, Hayakawa M, Kanai A, Shy Chen M, Alan Lewis R, Heckenlively J, Weleber RG, Traboulsi EI, Zhang Q, Xiao X, Kaiser-Kupfer M, Sergeev YV, Hejtmancik JF (2004). Bietti crystalline corneoretinal dystrophy is caused by mutations in the novel gene CYP4V2.. Am J Hum Genet.

[3] Lee J, Jiao X, Hejtmancik JF, Kaiser-Kupfer M, Gahl WA, Markello TC, Guo J, Chader GJ (2001). The metabolism of fatty acids in human Bietti crystalline dystrophy.. Invest Ophthalmol Vis Sci.

[4] Wilson DJ, Weleber RG, Klein ML, Welch RB, Green WR (1989). Bietti's Crystalline Dystrophy: A Clinicopathologic Correlative Study. Arch Ophthalmol.

[5] Qi JH, Dai G, Luthert P, Chaurasia S, Hollyfield J, Weber BH, Stöhr H, Anand-Apte B (2009). S156C mutation in tissue inhibitor of metalloproteinases-3 induces increased angiogenesis.. J Biol Chem.

[6] Le Tien V, Atmani K, Querques G, Massamba N, Souied EH (2010). Ranibizumab for subfoveal choroidal neovascularization in Bietti crystalline retinopathy.. Eye (Lond).

[7] Atmaca LS, Muftuoglu O, Atmaca-Sonmez P (2007). Peripapillary choroidal neovascularization in Bietti crystalline retinopathy.. Eye (Lond).

[8] Gupta B, Parvizi S, Mohamed MD (2011). Bietti crystalline dystrophy and choroidal neovascularisation.. Int Ophthalmol.

[9] Fiser A, Sali A (2003). Modeller: generation and refinement of homology-based protein structure models.. Methods Enzymol.

[10] Summa CM, Levitt M (2007). Near-native structure refinement using in vacuo energy minimization.. Proc Natl Acad Sci USA.

[11] Laskowski RA, Rullmannn JA, MacArthur MW, Kaptein R, Thornton JM (1996). AQUA and PROCHECK-NMR: programs for checking the quality of protein structures solved by NMR.. J Biomol NMR.

[12] Wallner B, Elofsson A (2003). Can correct protein models be identified?. Protein Sci.

[13] Ashkenazy H, Erez E, Martz E, Pupko T, Ben-Tal N (2010). ConSurf 2010: calculating evolutionary conservation in sequence and structure of proteins and nucleic acids.. Nucleic Acids Res.

[14] Thompson JD, Higgins DG, Gibson TJ (1994). CLUSTAL W: improving the sensitivity of progressive multiple sequence alignment through sequence weighting, position-specific gap penalties and weight matrix choice.. Nucleic Acids Res.

[15] Henikoff S, Henikoff JG (1992). Amino acid substitution matrices from protein blocks.. Proc Natl Acad Sci USA.

[16] Gromiha MM (2005). A statistical model for predicting protein folding rates from amino acid sequence with structural class information.. J Chem Inf Model.

[17] Shen B, Vihinen M (2003). RankViaContact: ranking and visualization of amino acid contacts.. Bioinformatics.

[18] Heinig M, Frishman D (2004). STRIDE: a web server for secondary structure assignment from known atomic coordinates of proteins.. Nucleic Acids Res.

[19] Baker NA, Sept D, Joseph S, Holst MJ, McCammon JA (2001). Electrostatics of nanosystems: application to microtubules and the ribosome.. Proc Natl Acad Sci USA.

[20] Guymer RH, Héon E, Lotery AJ, Munier FL, Schorderet DF, Baird PN, McNeil RJ, Haines H, Sheffield VC, Stone EM (2001). Variation of codons 1961 and 2177 of the Stargardt disease gene is not associated with age-related macular degeneration.. Arch Ophthalmol.

[21] Leu J, Schrage NF, Degenring RF (2007). Choroidal neovascularisation secondary to Best's disease in a 13-year-old boy treated by intravitreal bevacizumab.. Graefes Arch Clin Exp Ophthalmol.

[22] Rhee DY, Reichel E, Rogers A, Strominger M (2007). Subfoveal choroidal neovascularization in a 3-year-old child with North Carolina macular dystrophy.. J AAPOS.

